# Upper-Extremity Musculoskeletal Disorders and Their Associated Factors Among Diabetes Mellitus Patients Attending at Felege Hiwot Comprehensive Specialized Hospital, Bahir Dar, Northwest Ethiopia: Cross-Sectional Study

**DOI:** 10.3389/fendo.2022.856521

**Published:** 2022-04-26

**Authors:** Assefa Gebeyehu Muluneh, Kedir Sany Adem, Jemal Suleyman Dawud, Alemu Kassaw Kibret, Melisew Mekie Yitayal, Getachew Azeze Eriku

**Affiliations:** ^1^ Department of Physiotherapy, School of Medicine, College of Medicine and Health Sciences, Bahir Dar University, Bahir Dar, Ethiopia; ^2^ Department of Physiotherapy, School of Medicine, College of Medicine and Health Sciences, University of Gondar, Gondar, Ethiopia

**Keywords:** diabetes mellitus, upper extremity, musculoskeletal disorders, Ethiopia, musculoskeletal complications

## Abstract

**Background:**

Globally, diabetes mellitus (DM) is a major public health, causing functional disability among those affected. Among the common diabetes mellitus-related complications, musculoskeletal disorders had a significant negative impact on the social health quality of life and productivity of individuals. Evidence in this regard, however, is scarce in Ethiopia. Therefore, this study aimed at determining the prevalence of musculoskeletal disorders and identifying factors associated in people with diabetes in Felege Hiwot Comprehensive Specialized Hospital, northwest Ethiopia.

**Methods:**

A cross-sectional study design was employed at Felege Hiwot Comprehensive Specialized Hospital from June 01 to August 30, 2020, among 413 participants. A systematic random sampling was employed to select the study participants. A structured, pretested questionnaire was used to collect data on socio-demographic, clinical, and lifestyle characteristics. Musculoskeletal disorders were assessed through clinical and physical examination. A logistic regression model was used to explore factors associated with musculoskeletal disorders.

**Result:**

The prevalence of musculoskeletal disorders among diabetes mellitus patients was 24% (95% CI 20, 28.3). In logistic regression, factors such as being female, older age, occupation (farmer, merchant, and retired), and long duration of diabetes were significantly associated with musculoskeletal disorders.

**Conclusion:**

Nearly one quarter of the study participants had musculoskeletal disorders. Special attention should be given for those individuals living with diabetes mellitus, particularly for those who are female, are older, and had a prolonged history of diabetes mellitus.

## Introduction

Diabetes mellitus (DM) is a chronic and progressive metabolic disorder characterized by persistent hyperglycemia, which leads to morbidity and mortality due to secondary microvascular and macrovascular complications (1–3). Nowadays, it is one of the most important public health challenges (4, 5) and the fifth leading cause of death globally (6). Approximately 463 million people were affected by DM worldwide (7). It has been estimated that the number will increase to 700 million by the year 2045 (7, 8). Ethiopia is one of the first four countries in sub-Saharan Africa (SSA) to have a higher number of diabetes patients (5.2%) (9).

Although the pathophysiological mechanisms remain unclear, DM can lead to various musculoskeletal complications (2, 10) involving joints, soft tissues, muscles, nerves, and tendons (11). Musculoskeletal disorders are generally not diabetes mellitus specific and can also be seen in the general population. However, its incidence has increased significantly in patients with diabetes mellitus (10). It causes chronic irreversible damage and destructive changes in musculoskeletal and connective tissues associated with severity, duration of diabetes mellitus, and failure in controlling the blood glucose level (11). Evidence suggests that hyperglycemia may accelerate non-enzymatic glycosylation and abnormal collagen deposition in per-articular connective tissues that further alter the structural matrix and mechanical properties of the musculoskeletal system (12). In turn, connective tissue disorders, neuropathy, or micro- and macrovascular complications could have a synergic effect on the development of musculoskeletal complications associated with diabetes mellitus (13). Adhesive capsulitis of shoulder (AC), carpal tunnel syndrome (CTS), Dupuytren’s contracture (DC), trigger finger (TF), De Quervain’s contracture (DQC), and limited joint mobility (LJM) are those more commonly occurring in patients with diabetes mellitus (14–17).

Musculoskeletal disorders can cause pain (18, 19) as well as impairments in the hand and shoulder (20) resulting in loss of body function (11) and activity limitation (21), which is supported by the study conducted in Pakistan that revealed that advanced age, long duration of DM, and female gender were risk factors for shoulder pain and disability (22). It has a significant economic impact on the community due to direct medical and other related costs and disability which results in poor quality of life and health status of individuals with diabetes mellitus (19, 23). Furthermore, it interferes with diabetic care follow-up, work ability, and productivity of the workers (24).

Although the existing literature shows high burden of DM and associated MSKD globally, there is scarcity of evidence in our local arena and little attention is given by the government and other stakeholders. Thus, affected patients may have a decrease in afunctional ability, an increased sedentary lifestyle, and a poor quality of life (12). Therefore, it is important to identify musculoskeletal complications related to diabetes at an early stage to ensure quality of life and prevent long-term disability (25).

Despite the fact that evidence supports the inclusion of physical examination in the evaluation of diabetes mellitus patients, it has yet to be implemented in the national guidelines. Therefore, the study aimed at determining the prevalence and factors associated with MSKD in patients with diabetes attending at the Felege Hiwot Comprehensive Specialized Hospital, Bahir Dar, northwest Ethiopia. Besides, conducting the research may offer basic evidence for any interventions aimed at preventing and treating MSKD early and improving their quality of life, and designing effective strategies toward tackling preventable DM-related mortality and morbidity.

## Materials and Methods

### Study Design, Period, and Setting

This institution-based cross-sectional study was conducted from June 01 to August 31, 2020, in Felege Hiwot Comprehensive Specialized Hospital, Bahir Dar city, northwest Ethiopia. Bahir Dar City (the capital of Amhara regional state) is located around 560 km northwest of Addis Ababa (the capital of Ethiopia). Felege Hiwot Comprehensive Specialized Hospital (FHCSH) is one of the main hospitals in Bahir Dar town in Bahir Dar city which provides tertiary-level healthcare services as a major referral center for more than 5 million people from six different zones (Bahir Dar city administration, West Gojjam, east Gojjam, Awi, South and Central Gondar zones) in the Amhara region. The FHSH has a chronic illness outpatient department for the follow-up service for chronic disease including diabetes mellitus.

### Study Population and Eligibility Criteria

The study population for this study was all diabetes patients (both types of DM) who were attending their follow-up at the FNCSH chronic clinic. Diabetes mellitus patients with locomotive challenges and those who were visually impaired, mentally impaired, poststroke, pregnant, and with any known history of trauma and surgery were excluded.

### Sample Size Determination and Sampling Technique

A sample size was calculated using a single population proportion formula on the following assumptions: considering a 95% level of confidence, 5% precision, 10% non-response rate, and a 42% prevalence of MSDs among diabetes mellitus (26). Thus, $n=(\frac{Z\alpha /{2)}^{2}p*\left(1-p\right)}{{\left(d\right)}^{2}}=\frac{{\left(1.96\right)}^{2}*0.42\left(1-0.42\right)}{\left(0.05\right)2}=375$ where n = required sample size, α = level of confidence, z = standard normal distribution curve value for (% confidence level = 1.96, p = proportion of MSKD among DM patients, and d = margin of error. Finally, considering a 10% of non-response rate, the minimum adequate sample size was 413.

A 2-month report of DM patients attending the chronic follow-up clinic was obtained from the hospital record. According to the report obtained, about 75 to 85 DM patients visit the chronic OPD in FHCSH every day. Thereafter, the skipping interval (Kth) was calculated by dividing the 2-month report to the calculated sample size (1,360/422), resulting in 3. The study participants were selected every 3rd interval. The first case was selected randomly using a lottery method. Finally, a systematic random sampling technique was used to select all eligible diabetic patients.

### Data Collection Tools and Procedures

The data collection tool was developed from different literature (27–29). A structured, pretested, and interviewer-administered questionnaire and physical examination were used to collect the data.

The questionnaire consisted of two parts: the first part consisted of socio-demographic data, clinical factors, and lifestyle factors. The socio-demographic characteristics included age, sex, educational status, occupational status, family income, and BMI. The clinical factors included type of DM, duration of DM, medication, and presence of microvascular and macrovascular complications. The lifestyle factors included alcoholic, smoking, and physical activity status. BMI was calculated using the formula weight/height squared (k/m^2^). Four BSc physiotherapists and one MSc physiotherapist were recruited for data collection and supervision, respectively.

The second part of the questionnaire was diagnosis the MSKD. The diagnosis of musculoskeletal disorders was made health professional using clinical and physical examinations. The following criteria were used to identify all the musculoskeletal disorders.

Musculoskeletal disorders (AC, RCT, CTS, DQT, DC, LJM, and TF) were assessed by physiotherapists who received adequate training from the primary investigators based on participant’s history, medical chart, and standardized physical examination using special tests for each disorder and investigations if needed.

Adhesive capsulitis of shoulder was considered positive with patients having more restricted shoulder movement (equal restriction both passive and active movement) than normal in at least three planes (external rotation > abduction > internal rotation); unilateral pain for more than 1 month and inability to lie on the affected shoulder were diagnosed as adhesive capsulitis of shoulder (27, 30).

Limited joint mobility was considered present if one or more interphalangeal or metacarpophalangeal joints failed to make a contact when a patient was asked to oppose the palmar surfaces of the fingers in a praying position with the wrist maximally flexed, evaluated by the patient’s “prayer sign”) (27, 28, 31).

Rotator cuff tendinitis was considered positive with the presence of tenderness on the lateral side of the head of humerus below the acromion. Active abduction of the arm induced severe pain, especially in the arc between 60° and 120°.

Carpal tunnel syndrome was considered if there was a dull, aching discomfort in the hand, forearm, or upper arm, paresthesia in the hand, dryness of the hand, increase in symptoms during sleep, and improvement by shaking the wrist. A pinprick test for Tinel’s and Phalen’s signs was also performed on each patient suggestive of CTS (27, 32).

The Dupuytren’s contracture was diagnosed if there was a palmar or digital nodule, tethering of the palmar or digital skin, a peritendinous band, and a digital flexion contracture (25, 27, 33).

Trigger finger or tenosynovitis was diagnosed by palpating a nodule or thickened flexor tendon with locking happening in extension and flexion of any fingers (28, 34, 35).

De Quervain’s contracture was considered positive with the presence of pain and tenderness over the radial styloid with a positive Finkelstein maneuver (36, 37).

Musculoskeletal disorders: if one or more musculoskeletal disorders are present (Yes) (38).

Body mass index (BMI): it is the ratio of weight in kilogram over height in meter square: underweight = <18.5 kg/m^2^, normal = 18.5–24.9 kg/m^2^, overweight = 25.0–29.9 kg/m^2^, and obese = ≥30 kg/m^2^ (39). The height of the patients was measured without the footwear to the nearest 0.5 cm, and body weight was measured in light clothing on a level balance. BMI was calculated as weight (kg) divided by height (m^2^).

Smoking habit: a participant who smokes daily and occasionally (less than daily) per week is assumed to possess smoking (40).

Physically active: performing any moderate intensity physical activity including walking for at least 150 min per week (41).

### Data Quality Assurance

The questionnaire was first prepared in English and translated to the local language (Amharic) and back to English to keep its consistency, and readjustment of inconsistent and inaccurate data was done accordingly. A pretest was done on 5% of the sample size at the University of Gondar Comprehensive Specialized Hospital, Gondar, Ethiopia, to check the response and language clarity. A 1-day training was given by the principal investigators for the data collectors and the supervisor. The questionnaire was checked for completeness and consistency daily by the study supervisor and principal investigator.

### Data Processing and Analysis

Data were checked, coded, and entered into EPI INFO version 8.1 and exported into SPSS version 23.0 for analysis. Descriptive statistics like percentage, frequency, mean, standard deviations, tables, and graphs were used to present the characteristics of study participants. The binary logistic regression model was fitted to identify factors for MSKD among DM patients. All explanatory variables having a p-value of ≤0.2 in the bivariable analysis were included in the multivariable logistic regression analysis to handle the effect of possible cofounders and to identify factors associated with outcome variables. Then, the level of significance was declared based on the adjusted odds ratio (AOR) with 95% of CI at a p-value of ≤0.05.

### Ethical Consideration

Ethical clearance was obtained from the ethical review committee of the school of medicine under the delegation of the University of Gondar ethical review board (reference number 1925/03/2020). An official letter of support was received from the University of Gondar and was sent to the clinical director of the Felege Hiwot Comprehensive Specialized Hospital. Before the data collection, the purpose of the study, the potential indirect benefit, and the right to refuse were explained to each study participant. Finally, a written informed consent was taken from each participant. The confidentiality of the information was assured throughout the data collection.

## Result

### Participant’s Socio-Demographic Characteristics

A total of 413 study participants were included with a response rate of 100%. The mean age of the participants was 45.37 (SD ± 15.327), and 54.7% of the participants were less than 50 years of age. More than half (53.5%) and two-thirds (67.6%) of the study participants were male and in marital union, respectively. Regarding the place of residence, about 71.2% of the study participants were from urban areas and one-third of them were unable to read and write (**Table 1**).

**Table 1:** Socio-demographic characteristics of participants (N = 413).

Variables	Category	Frequency (%)
Age	<50 years	226 (54.7)
	>50 years	187 (45.3)
Sex	Male	221 (53.5)
	Female	192 (46.5)
Place of residence	Urban	294 (71.2)
	Rural	119 (28.8)
Marital status	Never married	134 (32.4)
	Married	279 (67.6)
Educational status	Unable to read and write	142 (34.4)
	Primary education	100 (24.2)
	Secondary education	57 (13.8)
	Diploma and above	114 (27.6)
Occupation	Housewife	90 (21.8)
	Farmer	73 (17.7)
	Merchant	95 (23.0)
	Government employee	122 (29.5)
	Retired	33 (8)
Family income	<600	118 (28.6)
	600–1,000	115 (27.8)
	1,001–3,000	81 (19.6)
	>3,000	99 (24)
BMI	Underweight	28 (6.8)
	Normal	245 (59.3)
	Overweight	110 (26.6)
	Obese	30 (7.3)

### Clinical Characteristics of the Study Participants

More than half of the study participants have type 2 DM, and nearly two-thirds of the participants were insulin dependent. Regarding vascular complications, 17.7% of participants have had retinopathy while 1.7% of diabetic patients had coronary artery disease (**Table 2**).

**Table 2:** Clinical-related characteristics of diabetic patients in FHCSH, Bahir Dar, North West Ethiopia, 2020 (n = 413).

Variables	Category	Frequency (%)
Duration of DM	<5 years	142 (34.0)
	5 to 9 years	147 (35.6)
	≥10 years	124 (30.4)
Types of DM	Type 1	192 (46.5)
	Type 2	221 (53.5)
Medication	Insulin	245 (59.3)
	Medication	161 (39)
	Both	7 (1.7)
Microvascular complication	Yes	91 (22)
	No	322 (78)
Macro vascular complications	Yes	9 (2.2)
	No	404 (97.8)

### Lifestyle Characteristics of the Study Participants

Majority of the participants never smoke cigarette (95.2%) and do not drink alcohol (92.5%). Slightly more than half of the participants (53.3%) did not have the practice to engage in moderate physical activity (**Table 3**).

**Table 3:** Lifestyle characteristics of participants (N = 413).

Variables	Category	Frequency (%)
Smoking habit	Yes	20 (4.8)
	No	393 (95.2)
Alcohol consumption	Yes	31 (7.5%)
	No	382 (92.5)
Physical activity	Yes	193 (46.7)
	No	220 (53.3)

### Prevalence of Musculoskeletal Disorders Among Diabetic Patients

**Figure 1:**
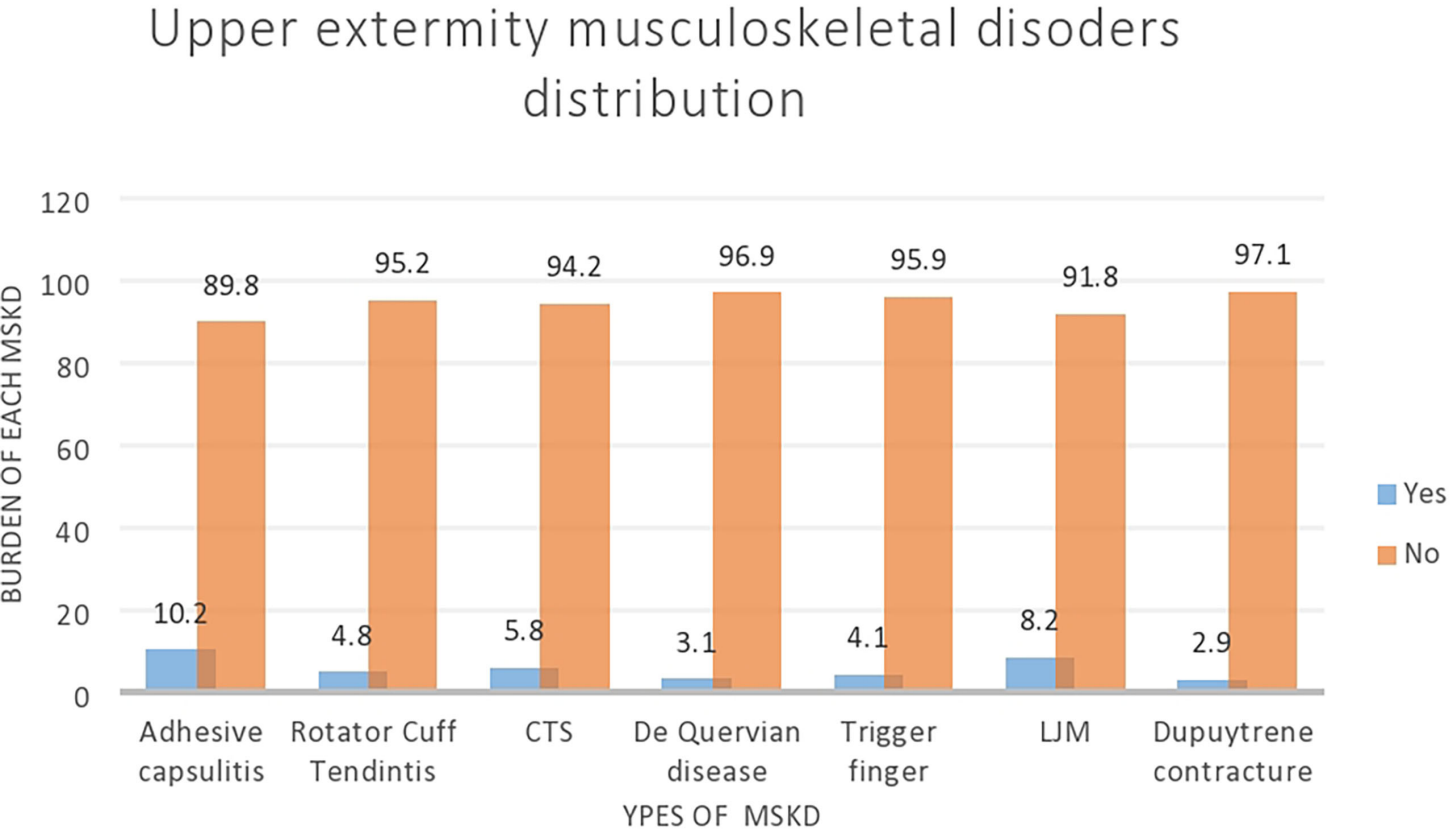
The upper extremity musculoskeletal disorders.

The prevalence of MSKD among patients with diabetes was 24% (95% CI: 19.9–28.0). The highest prevalence was for AC (10.2%) followed by LJM (8.2%) (**Figure 1**).

### Factors Associated With MSDs Among Diabetic Patients

Age, sex, marital status, place of residence, BMI, physical activity, duration of DM, type of DM, and presence of microvascular and macrovascular complications were entered into multivariable analysis. Age, sex, occupation, and higher duration of DM were significantly associated with MSKD.

The odds of having musculoskeletal disorders for DM patients aged 50 and above were 4.64 times higher compared to the age group under 50 years of age [AOR = 4.64 (95% CI: 2.45–8.89)]. Female DM patients were 2.05 times more likely to have musculoskeletal disorders than male DM patients [AOR = 2.05 (95% CI: 1.05, 4.03)]. We also found MSKD occupations: farmer [AOR = 0.21 (95% CI: 0.52–0.84), merchant [AOR = 2.55 (95% CI: 1.09–5.95)], and retired [AOR = 3.14 (95% CI: 1.09–9.03)]. Participants who had 10 years or above duration of DM had 4.3 times the odds of having MSKD as compared to participants who had less than 5 years of duration of DM [AOR = 4.3 (95% CI: 2.11–8.84)] **(** **Table 4** **).**

**Table 4:** Bivariate and multivariate logistic regression analyses of factors associated with MSKD among diabetic patients at FHCSH, Bahir Dar, Northwest Ethiopia, 2020 (n = 413).

Variables	Categories	No (%)	MSKD		COR (95%)	AOR (95%)	p-value
			Yes	No			
**Age**	<50 years	226 (54.7)	12 (5.3)	214 (94.7)	1	1	1
	≥50 years	187 (45.3)	87 (46.5)	100 (53.5)	7.5 (4.38, 12.97)	4.64 (2.45, 8.89)	0.000**
**sex**	Male	221 (53.5)	43 (19.5)	178 (80.5)	1		
	Female	192 (46.5)	56 (29.2)	136 (70.8)	1.8 (1.14, 2.84)	2.05 (1.05, 4.03)	0.035**
**Marital status**	Never married	134 (32.4)	38 (28.4)	96 (71.6)	1	1	
	Married	279 (67.6)	61 (24.7)	218 (75.3)	0.7 (0.44, 1.13)	0.78 (0.418, 1.43)	0.419
**Place of residence**	Urban	294 (71.2)	76 (25.9)	218 (74.1)	1.9 (1.131,3.423)	0.63 (0.29, 1.31)	0.216
	Rural	119 (28.8)	23 (19.3)	96 (80.7)	1	1	
**Occupation**	Housewife	90 (21.8)	28 (31.1)	62 (68.9)	1	1	
	Farmer	73 (17.7)	3 (4.1)	70 (95.9)	0.09 (0.027, 0.328)	0.21 (0.52,0.84)	0.027
	Merchant	95 (23.0)	28 (29.5)	67 (70.5)	0.92 (0.494, 1.733)	2.55 (1.09,5.95)	0.031
	Government employee	122 (29.5)	20 (16.4)	102 (83.6)	0.43 (0.226, 0.836)	0.98 (0.40,2.29)	0.939
	Retired	33 (8)	20 (60.6)	13 (39.4)	3.4 (1.48,7.80)	3.14 (1.09,9.03)	0.034
**BMI**	Underweight	28 (6.8)	4 (14.3)	24 (85.7)	1	1	
	Normal	145 (59.3)	47 (19.2)	198 (19.2)	1.06 (0.38, 2.94)	1.22 (0.36,4.12)	0.749
	Overweight	110 (26.6)	33 (30)	77 (70)	2.05 (0.72,5.87)	1.44 (0.39, 5.27)	0.582
	Obese	30 (7.3)	15 (50)	15 (50)	4.03 (1.20, 13.45)	2.82 (0.66,11.97)	0.160
**Physical activity**	Yes	193 (46.7)	29 (15)	164 (85)	1		
	No	220 (53.3)	70 (31.8)	150 (68.2)	2.5 (1.53, 4.02)	1.1 (0.58, 1.95)	0.820
**Duration of DM**	<5 years	142 (34)	6 (4.2)	136 (95.8)	1	1	
	5 to 9 years	147 (35.6)	23 (15.6)	124 (84.4)	1.46 (0.73,3.89)	0.87 (0.40,1.86)	0.724
	≥10 years	124 (30.4)	70 (56.5)	54 (43.5)	7.4 (3.93, 13.83)	4.3 (2.11, 8.84)	0.000**
**Types of DM**	Type 1	192 (46.5)	22 (11.5)	170 (88.50	1	1	
	Type 2	221 (53.5)	77 (34.8)	144 (65.2)	2.9 (1.80, 4.84)	0.7 (0.34,1.45)	0.344
**Microvascular complication**	Yes	90 (21.8)	42 (46.2)	49 (53.8)	0.35 (0.21, 0.57)	0.67 (0.36, 1.23)	0.199
	No	322 (78.2)	57 (17.7)	265 (82.3)	1	1	
**Macrovascular complication**	Yes	9 (2.2)	4 (44.4)	5 (55.6)	0.38 (0.10, 1.46)	0.67 (0.36,1.26)	0.626
	No	404 (97.8)	95 (23.5)	309 (76.5)	1	1	

The ** indicate the variables which have very significant association with the upper extremity Musculoskeletal disorders.

## Discussion

The purpose of this study was to find out the prevalence and associated factors of MSKD in patients with diabetes in the Felege Hiwot Comprehensive Specialized Hospital, Bahir Dar, northwest Ethiopia. In our study, the prevalence of MSKDs among DM patients was 24% which is higher than in those studies conducted in Ethiopia which is 16.6% (42), Netherlands 16.3% (43), India 19.8% (36), and Saudi Arabia 17.9% (44). Late diagnosis of DM, lack of awareness and screening practices, poor glycemic control, and/or inadequate clinical management of musculoskeletal complications could be attributed to the high burden of MSKD (45). In Ethiopia, a high proportion of diabetic patients had poor glycemic control (46, 47) and poor diabetic self-care practice (48).

However, this result is lower as compared to previous studies done in France at 28.6% (14), Morocco 34.4% (29), India 42.58% (27), Turkey 45.9% (49), Pakistan 46.7% (50), Jordan 69.5% (28), Canada 66% (51), Sweden 65% (52), and Iran 83.5% (53). This difference might be explained by various procedures used to measure the prevalence and the type of DM (either type I only or type II or both). This might be also justified as difference in socioeconomic status and life style. Besides, some studies measured only the shoulder or hand separately to assess MSKD whereas others looked at the whole body including lower-extremity disorders. Individual differences in the provision of healthcare as well as adherence to management to ensure good diabetic control and keep musculoskeletal function supple by modifying life style, engaging in regular physical activity, and strictly adhering to prescribed diet management could also be reasons for non-compliance.

Adhesive capsulitis of the shoulder (10.2%) was the most commonly reported MSKD in our study followed by limited joint mobility 8.2%, carpal tunnel syndrome 5.8%, rotator cuff tendinitis 4.8%, trigger finger 4.1%, De Quervain’s tenosynovitis 3.1%, and Dupuytren’s contracture 2.9%, which is consistent with a previous study done in Pakistan (54) and Iran (38) in which the proportion of adhesive capsulitis of shoulder was 10.9% and 8.79%, respectively. However, the magnitude was higher than that in a study conducted in Saudi Arabia (44).

In our study, the results of multivariate analysis revealed that being female, older age, occupation (farmer, merchant, and being retired) and long duration of DM were factors significantly associated with the presence of one or more MSKD.

Increasing age is a significant risk factor in developing musculoskeletal disorders. Our study found that older adults over the age of 50 were 4.64 times more likely to develop one or more MSKDs than younger participants under the age of 50. This finding is consistent with the studies conducted in Iran (53), Jordan (28), Turkey (49), and India (27). The possible explanation could be that skeletal muscle mass and strength decline with age due to a reduction in the number of muscle fibers and cellular and molecular changes that reduce the force-generated process (19, 55, 56). Loss of bone, articular cartilage degradation, degeneration, and narrowing of the interverbal discs are the primary features of aging. They go through significant biomechanical alternations that directly compromise their biomechanical function, which contributes to the development of MSKD, which also contributes to pain and disability (19, 57).

In our study, MSKD was observed more frequently in female (29.2%) participants as compared with male participants. Hence, female diabetic participants were two times more likely to develop musculoskeletal manifestations as compared to male participants. This was in line with the findings reported from studies done in Ethiopia (42) and Jordan. Consistent with this finding, the review literature revealed that females had a significantly higher incidence of various types of musculoskeletal complications than males (10). Females may have poor glycemic control, less engagement in physical activity, and less adherence to therapy than males, which may contribute to the discrepancy in the findings.

Regardless of the evidence associating occupation with the presence of MSKD in diabetes patients, farmers, merchants, and retirees were associated with the presence of MSKD. Farmers were less likely than housewives to have MSKD. One possible explanation is that farmers were more physically active than housewives, which reduced the likelihood of developing musculoskeletal complications. Merchants, on the other hand, are more likely to have MSKD than housewives. This could also be that the job may contribute for the sedentary lifestyle of merchants. Participants who had retired from their occupation, on the other hand, were three times more likely to have one or more MSKD than housewives. One plausible explanation is that the likelihood of having one or more MSKD increases proportionally with age according to the finding from our study.

The presence of one or more MSKD was significantly associated with the duration of DM in our study. Study participants living with DM for more than 10 years had 4.3 times higher odds of developing MSDs than those participants with a diabetes duration of less than 5 years. The findings in our study were supported by studies done in Jordan (28),Turkey (49), and India (27). The most likely explanation is that musculoskeletal changes may progress in a proportional manner as the duration of diabetes increases.

Our study has certain potential shortcomings that point to the coming research. First, it was conducted in a single health institution with a homogenous study population, which may limit our ability to conclude. Second, our study is cross-sectional, which limits our capacity to establish a casual relation between explanatory and response variables. Finally, due to the incapability of imaging and laboratory study to diagnose prevalent MSKD such as osteoarthritis, we do not include them.

## Conclusion

In this study, approximately one-quarter of the study participants had MSKD. Being older, female sex, retired, occupation, and having diabetes for a longer period of time were significantly associated with the occurrence of one or more musculoskeletal disorders in patients with diabetes.

## Data Availability Statement

The original contributions presented in the study are included in the article/supplementary material. Further inquiries can be directed to the corresponding author.

## Ethics Statement

The studies involving human participants were reviewed and approved by the School of Ethical Review Committee. The patients/participants provided their written informed consent to participate in this study.

## Author Contributions

All authors contributed to the data analysis, drafting, or revising of the article, gave final approval of the manuscript to be published, and agree to be accountable for all aspects of the work.

## Funding

The research project was funded by University of Gondar.

## Conflict of Interest

The authors declare that the research was conducted in the absence of any commercial or financial relationships that could be construed as a potential conflict of interest.

## Publisher’s Note

All claims expressed in this article are solely those of the authors and do not necessarily represent those of their affiliated organizations, or those of the publisher, the editors and the reviewers. Any product that may be evaluated in this article, or claim that may be made by its manufacturer, is not guaranteed or endorsed by the publisher.

## Abbreviations

AOR, adjusted odds ratio; CI, confidence interval; COR, crude odds ratio; DM, diabetes mellitus; MSKD, musculoskeletal disorders; SD, standard deviations; VAS, visual analog scale.
